# Effects of Visual Speech on Early Auditory Evoked Fields - From the Viewpoint of Individual Variance

**DOI:** 10.1371/journal.pone.0170166

**Published:** 2017-01-31

**Authors:** Izumi Yahata, Tetsuaki Kawase, Akitake Kanno, Hiroshi Hidaka, Shuichi Sakamoto, Nobukazu Nakasato, Ryuta Kawashima, Yukio Katori

**Affiliations:** 1 Department of Otolaryngology-Head and Neck Surgery, Tohoku University Graduate School of Medicine, Sendai, Miyagi, Japan; 2 Laboratory of Rehabilitative Auditory Science, Tohoku University Graduate School of Biomedical Engineering, Sendai, Miyagi, Japan; 3 Department of Audiology, Tohoku University Graduate School of Medicine, Sendai, Miyagi, Japan; 4 Department of Functional Brain Imaging, Institute of Development, Aging and Cancer, Tohoku University, Sendai, Miyagi, Japan; 5 Research Institute of Electrical Communication, Tohoku University, Sendai, Miyagi, Japan; 6 Department of Epileptology, Tohoku University Graduate School of Medicine, Sendai, Miyagi, Japan; Harvard Medical School, UNITED STATES

## Abstract

The effects of visual speech (the moving image of the speaker’s face uttering speech sound) on early auditory evoked fields (AEFs) were examined using a helmet-shaped magnetoencephalography system in 12 healthy volunteers (9 males, mean age 35.5 years). AEFs (N100m) in response to the monosyllabic sound /be/ were recorded and analyzed under three different visual stimulus conditions, the moving image of the same speaker’s face uttering /be/ (congruent visual stimuli) or uttering /ge/ (incongruent visual stimuli), and visual noise (still image processed from speaker’s face using a strong Gaussian filter: control condition). On average, latency of N100m was significantly shortened in the bilateral hemispheres for both congruent and incongruent auditory/visual (A/V) stimuli, compared to the control A/V condition. However, the degree of N100m shortening was not significantly different between the congruent and incongruent A/V conditions, despite the significant differences in psychophysical responses between these two A/V conditions. Moreover, analysis of the magnitudes of these visual effects on AEFs in individuals showed that the lip-reading effects on AEFs tended to be well correlated between the two different audio-visual conditions (congruent vs. incongruent visual stimuli) in the bilateral hemispheres but were not significantly correlated between right and left hemisphere. On the other hand, no significant correlation was observed between the magnitudes of visual speech effects and psychophysical responses. These results may indicate that the auditory-visual interaction observed on the N100m is a fundamental process which does not depend on the congruency of the visual information.

## Introduction

The information of visual speech (the moving image of the speaker’s face uttering speech sound) presented with speech sound is known to help speech perception under conditions of impaired hearing, such as in noisy environments and/or in subjects with impaired hearing (lip-reading effects) [1, 2]. This lip-reading effect is usually useful in perceiving congruent audio-visual (A/V) information, such as watching the speaker’s face when listening to speech, but may also be influenced by incongruent A/V information. For example, if the /ba/ sound (auditory stimulus) is presented with the speaker’s face uttering the /ga/ sound (incongruent visual stimulus) simultaneously, the presented /ba/ sound is often perceived as /da/; i.e., the auditory perception may be modified by the incongruent visual information. This lip-reading effect under incongruent condition, which is known as the “McGurk effect,” is important evidence that the lip-reading effect can be observed not only in subjects with impaired hearing but also in subjects with normal hearing [3]. Therefore, it is important to understand how the perception caused by auditory input is affected by the visual input presented simultaneously in order to understand the underlying mechanism of lip-reading.

Hemodynamic brain imaging such as functional magnetic resonance (MR) imaging and positron emission tomography has established the important role of the left superior temporal sulcus (STS) in auditory-visual integration in relation to human lip-reading effects [4–10]. Moreover, possible modulation effects of visual stimuli (lip-reading effects) were indicated at earlier processing sites such as the superior temporal plane and superior temporal gyrus as well, in which the primary and/or secondary auditory cortex is thought to be located [6, 11–14]. The lip-reading effects seen in the auditory cortices have also been observed in auditory evoked responses originating from the auditory cortices using electroencephalography and magnetoencephalography (MEG). In most such studies, the latencies of the N100 responses (or N100m in MEG) to speech sound, which are one of the most stable evoked waves occurring with a post-stimulus latency of approximately 100 ms, are shortened by the presentation of visual speech; i.e., the visual speech stimuli presented synchronously with auditory speech could speed up (facilitate) the processing of auditory speech at the level of the auditory cortex [8, 15–17].

This visual effect on N100m can be observed with any type of visual speech presented synchronously with speech sound regardless of congruency between the auditory and visual inputs. The magnitude of visual effects are also considerably affected by the visual predictability (ease of recognition of the speech information based only on visual clues) or attention [15, 17].

Such visual speech effects on N100m have so far been assessed based on comparisons between groups with different A/V conditions. However, considering that the magnitudes of visual speech effects are known to somewhat vary among the subjects [18], assessment of the visual effects in individuals may also be important.

Thus, the present study investigated the individual variation in the effects of lip reading on the latencies of N100m as well as psychophysical responses, by examining the lip-reading effects in individual subjects under different visual conditions. The visual-speech effects of the known latency shortening of N100m is usually associated with amplitude reduction of the N100(m) responses as well [8, 15–17, 19–22]. However, the amplitude reduction of N100m may also be caused by the load of uncorrelated visual stimuli, not only by lip-reading effects; i.e., the perceptual load of visual information may generally reduce the auditory cortex responses, as a result of the possible cross-modal perceptual load effect [23]. Therefore, the present study particularly analyzed the visual effects on the N100m latency.

## Methods

### Subjects

Twelve healthy subjects participated in this study (9 males and 3 females, mean age 35.5 years), with normal hearing without any history of auditory diseases and/or neurological disorders. All subjects were native Japanese speakers and classified as right-handed with scores above +90 on the Edinburgh Handedness Inventory [24]. All procedures of the present study were approved by the ethical committee of Tohoku University Graduate School of Medicine, and written informed consent in accordance with the requirements of the ethical committee was obtained from each subject. All parts of the present study were performed in accordance with the guidelines of the Declaration of Helsinki (1991).

### A/V stimuli

**Fig 1:**
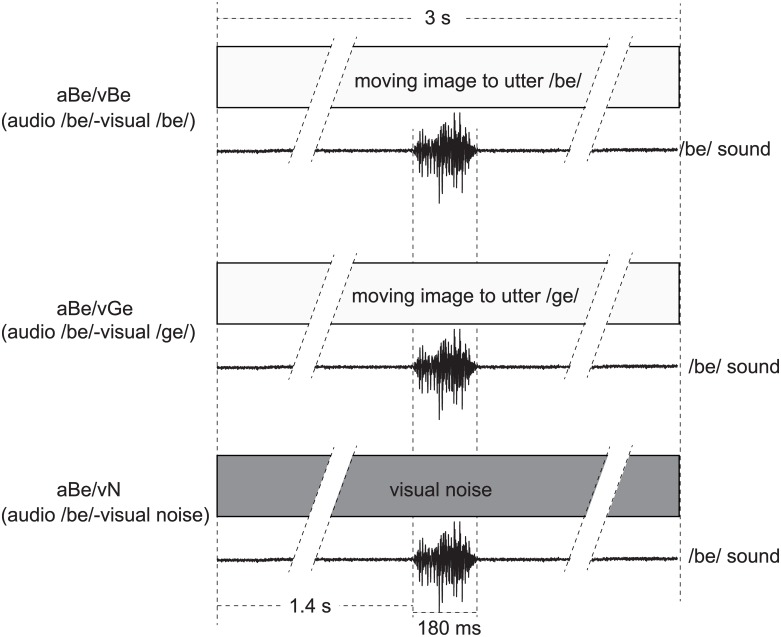
Schematic drawings of the three A/V stimuli used in the present study. aBe/vBe (audio /be/ and visual /be/), a monosyllabic sound /be/ spoken by a Japanese male speaker with the moving image of the same speaker’s face uttering /be/ (congruent visual stimulus); aBe/vGe (audio /be/ and visual /ge/), the same /be/ sound with the moving image of the same speaker’s face uttering /ge/ (incongruent visual stimulus); and aBe/vN (audio /be/ and visual /noise/), the same /be/ sound with visual noise created by applying a strong Gaussian filter of a PC software (Adobe^®^ Photoshop) to a still image of the speaker’s face during the utterance of /be/. Total duration of each video clip was 3 s. The audio stimulus of the /be/ sound (duration about 180 ms) was presented starting at 1.4 s after the beginning of the visual stimulus. Audio stimuli were synchronized with the speaker’s mouth movement.

Auditory evoked fields (AEFs) in response to three different A/V stimuli were compared (Fig 1). The stimuli consisted of one acoustic stimulus, a monosyllabic sound /be/ (aBe) spoken by a Japanese male speaker, and three visual stimuli, the moving images of the same speaker’s face uttering /be/ (congruent visual stimulus, vBe) or /ge/ (incongruent visual stimulus, vGe), and visual/noise/ (vN) created by applying a strong Gaussian filter of a PC software (Adobe^®^ Photoshop) to a still image of the speaker’s face during the utterance of /be/. The duration of each video clip was 3 s. The audio stimulus was started 1.4 s after the beginning of the visual stimulus and lasted approximately 180 ms, so as to be synchronized with the speaker’s mouth movement to achieve the conditions of congruent A/V stimulus (audio /be/ and visual /be/, aBe/vBe) as well as incongruent A/V stimulus (audio /be/ and visual /ge/, aBe/vGe) (see Fig 1 for details).

### Stimulus presentation

Software Presentation^®^ (Neurobehavioral Systems, Inc., Berkeley, CA) was used to present the A/V stimuli. Visual stimuli were presented 35 cm in front of the participants in a magnetically shielded room using a video projector and a monitor. Audio stimuli at a sound pressure level of 80 dB were presented bilaterally through tube earphones (ER-3A^®^, Etymotic Research, Inc., Elk Grove Village, IL).

The three A/V stimuli (aBe/vBe, aBe/vGe, aBe/vN) were presented in random order with inter-stimulus interval of 3 s. Previous studies have examined the A/V effects by two types of comparisons, A only condition vs. A/V condition, and A only condition vs. (A/V condition—V only condition) [8, 15–17, 19–21]. In the present study, the vN was used as a control visual stimulus. Thus, responses for the A/active V condition were compared with those for A/still V (visual noise).

Each A/V stimulus was presented about 100 times. During the inter-stimulus interval of 3 s, a black screen with a small red cross, located near the “mouth” position in the moving image of the speaker’s face, was shown to fix the direction of the subject’s eyes. The subjects were asked to judge “what the A/V stimulus was heard as” and push one of three response buttons to maintain the attention level. The subjects were directed to push the first and second buttons, if the stimulus was heard as /de/ and /be/, respectively, and to push the third button if the stimulus was heard as other than /de/ and /be/.

### MEG recording

MEG was used to detect the AEFs using a 200-channel whole-head type axial gradiometer system (MEG vision PQA160C, Yokogawa Electric, Musashino, Tokyo, Japan) in a magnetically shielded room. The detailed conditions of the MEG system used in the study already described [25]. Briefly, the sensors were first-order axial gradiometers with a baseline of 50 mm and 15.5-mm diameter coils. The sensors were arranged in a uniform array over a helmet-shaped surface at the bottom of a dewar vessel. The centers of two adjacent coils were separated by a mean distance of 25 mm. The field sensitivity of the sensors (system noise) was 3 fT/Hz within the frequency range used. Real-time MEG monitoring of the occipital alpha rhythm confirmed that the AEFs were recorded only in the awake state. The MEG signal was band-pass filtered between 0.16 and 100 Hz, and sampled at 500 Hz.

Coils were attached to the head surface at 5 locations to act as fiduciary points with respect to the landmarks (nasion and preauricular points) and the position of the head within the helmet by passing currents through the coils and measuring the magnetic fields. In addition, the head shape of each participant was digitized using a three-dimensional digitizer (FastSCAN Cobra, Polhemus Inc., Colchester, VT) and co-registered with individual structural MR images acquired using a 3 T MR system (Achieva, Philips, Best, the Netherlands).

### Analysis

All MEG signals were continuously recorded during the entire experimental duration, and later analyzed (offline) using the built-in software in the MEG system (MEG Laboratory, Yokogawa Electric) to obtain AEFs to each A/V stimulus. To obtain N100m responses to monosyllabic audio stimuli under each visual condition, the data from 1100 ms before to 1500 ms after the onset of each visual stimulus were averaged using the baseline from 1100 to 0 ms before the onset about 100 times. In the following off-line analysis, the averaged data were digitally band-pass filtered from 2.0 to 45.0 Hz. The N100m response was visually identified as the first prominent peak at 80–140 ms after the onset, with the isofield map confirming downward current orientation. The locations of the signal sources of N100m were estimated using an equivalent current dipole (ECD) model using the best-fit sphere for each subject’s head. A single ECD model based on Sarvas law [26] in a spherical volume conductor was used to identify the sources of the magnetic signals. The locations in the right and left hemispheres of the signal source were separately analyzed. ECDs with goodness-of-fit value of 90% were superimposed on the individual three-dimensional MR images using a MEG-MR image coordination integration system and the measured responses were verified to originate from the auditory cortex.

The latencies of the N100m were compared for the three A/V stimuli. The latency of N100m in the maximum activity channel of each hemisphere as well as those of the averaged wave (root mean square [RMS] waves) were assessed by off-line analysis. Basically, the same effects were obtained from both analyses. However, considering the general stability of averaged data, data analyzed based on RMS waves are shown in the present paper.

Analysis of variance with SPSS version 21 (IBM, Armonk, NY) software and/or Statview version 5 software (SAS, Cary, NC) was used to evaluate significant differences in peak latency of the N100m. P<0.05 was considered to be significant.

## Results

### Psychophysical findings

**Fig 2:**
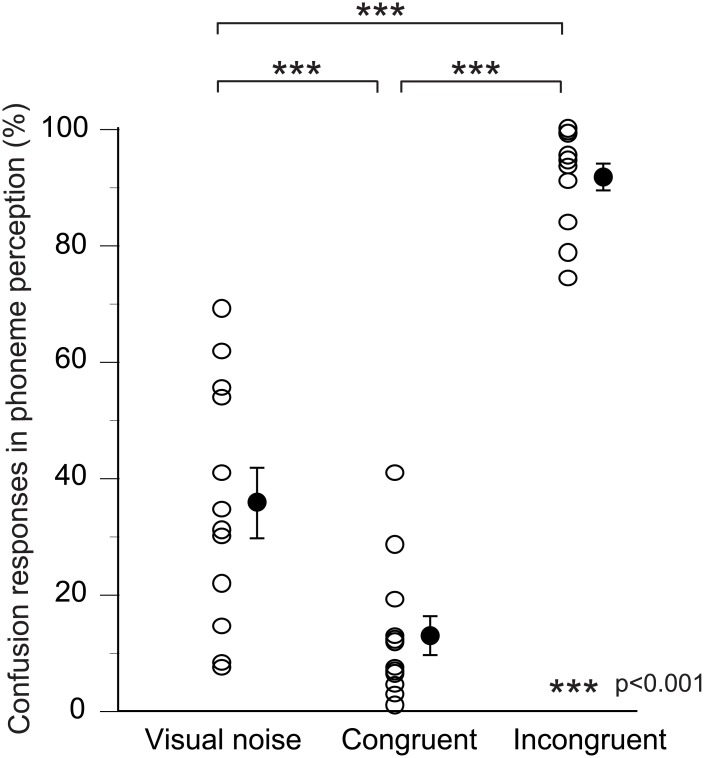
Psychophysical responses to the three A/V stimuli during the MEG measurements. Subjects were asked to judge “what the A/V stimulus was heard as” by pushing the response buttons. The rate (percentage) at which the presented /be/ sound was perceived as different to /be/ was plotted for the three different A/V conditions. Open circles indicate individual data. Average and standard error values are represented by filled circles and bars, respectively. Statistical significance of differences was determined by one-way repeated measures analysis of variance with Bonferroni post-hoc analysis. Asterisks indicate significant differences (p<0.001). As expected, the confusion response (the rate at which /be/ sound was perceived as different to /be/) was significantly higher under the incongruent McGurk visual condition (visual /ge/) and was significantly lower under the congruent visual condition (visual /be/).

In the present study, AEFs (N100m) in response to the audio /be/ were recorded under three different visual stimulus conditions, visual /be/, visual /ge/, and visual noise. As expected, the sounds were perceived differently according to the three visual conditions; i.e., the rate at which /be/ sound was perceived as different to /be/ was significantly higher under the incongruent McGurk visual condition (visual /ge/) and was significantly lower under the congruent visual condition (Fig 2). That is, our behavioral data strongly indicate that extensive lip-reading effects occurred under the present experimental conditions.

### Lip-reading effects on N100m

**Fig 3:**
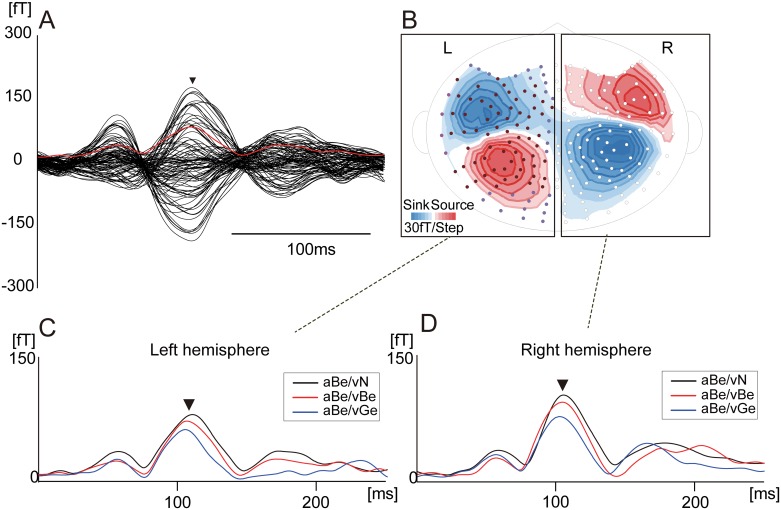
Typical examples of waveforms of the auditory evoked fields (AEFs) under the three A/V conditions (Subject 1). Asterisks in A, C, and D indicate N100m. A: Superimposed waveforms recorded from all sensors located in the right hemisphere (black line) and root mean square (RMS) waveform (red line). B: Iso-field map. C and D: RMS waveforms calculated from all sensors in the right (C) and left hemispheres (D) for the three A/V conditions. Black, red, and blue waveforms indicate aBe/vN, aBe/vBe, and aBe/vGe, respectively.

Typical examples of the RMS waveforms are shown for each hemisphere in Fig 3. Shortening of N100m latency associated with amplitude reduction was observed in both hemispheres.

**Fig 4:**
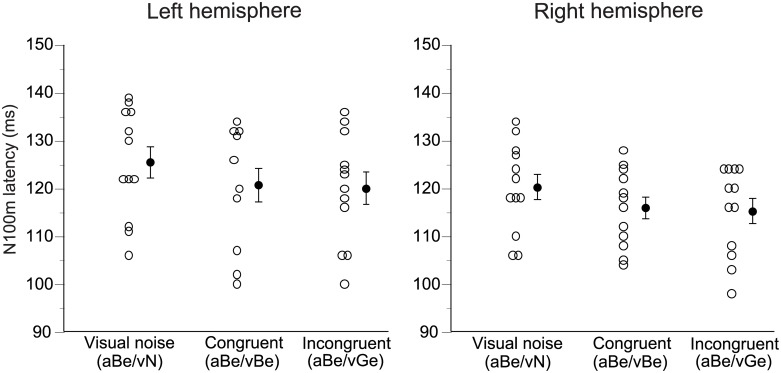
N100m latencies under the three A/V conditions. N100m RMS peak latencies of three A/V conditions are shown for the left (left column) and right hemispheres (right column). Open circles indicate individual data. Average and standard error values are represented by filled circles and bars, respectively. Analysis of variance of the N100m latencies for the three A/V conditions was conducted with factors of subject, hemisphere, and A/V condition. Only the A/V condition was significant (F = 30.0 (degree of freedom = 2), p < 0.001) without interaction between the hemisphere and A/V condition (F = 0.08 (degree of freedom = 2), p = 0.93). Multiple comparison procedures using Scheffe’s test showed that significant shortening of N100m latency compared with the control condition (visual noise) were observed in both congruent (aBe/vBe) (p < 0.001) and incongruent (aBe/vGe) A/V conditions (p < 0.001). N100m showed no significant differences between congruent and incongruent A/V conditions.

**Fig 5:**
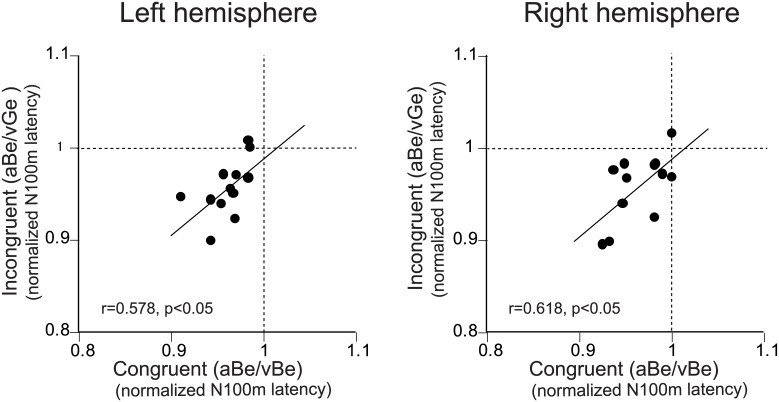
Relationship between lip-reading effects on N100m latencies under congruent and incongruent A/V conditions. Relationships between the normalized N100m latency in each subject for congruent (aBe/vBe) A/V stimuli (ratio between N100m latency under the aBe/vBe and aBe/vN conditions) and incongruent (aBe/vGe) A/V stimuli (ratio between N100m latency under the aBe/vGe and aBe/vN conditions) are represented for the left and right hemispheres. Thin line in each figure indicates the linear regression line.

**Fig 6:**
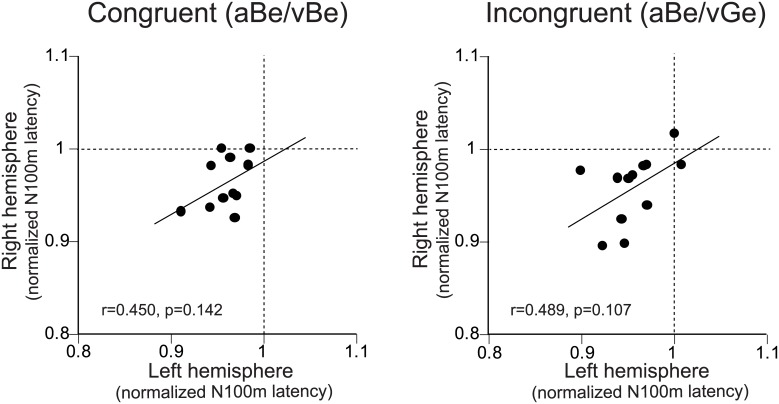
Relationship of lip-reading effects on N100m latencies between the right and left hemispheres. Normalized N100m latencies (ratio between N100m latencies under the aBe/vBe or aBe/vGe conditions and control [aBe/vN] conditions) in the right hemisphere are plotted as a function of those in the left hemisphere (left: congruent [aBe/vBe] A/V condition, right: incongruent [aBe/vGe] condition). Thin line in each figure indicates the linear regression line.

N100m RMS peak latencies under the three A/V conditions are shown in Fig 4. On average, significant shortenings of N100m latency compared with the control condition (visual noise) were observed under both congruent (aBe/vBe) and incongruent (aBe/vGe) A/V conditions. Conversely, N100m latencies showed no significant differences between congruent and incongruent A/V conditions. As shown, the N100m latencies were relatively varied among the subjects. Therefore, the effects of visual speech were analyzed on an individual basis in Figs 5 and 6.

The relationships of the magnitudes of visual speech effects between the incongruent and congruent visual conditions across the subjects are shown in Fig 5. Significant correlation between the lip-reading effects of congruent and incongruent visual information was observed. That is, larger lip-reading effects of congruent A/V stimuli tended to be associated with larger effects of incongruent stimuli. In contrast, the relationships of the magnitudes of visual speech effects between the right and left hemispheres were not significant (Fig 6).

**Fig 7:**
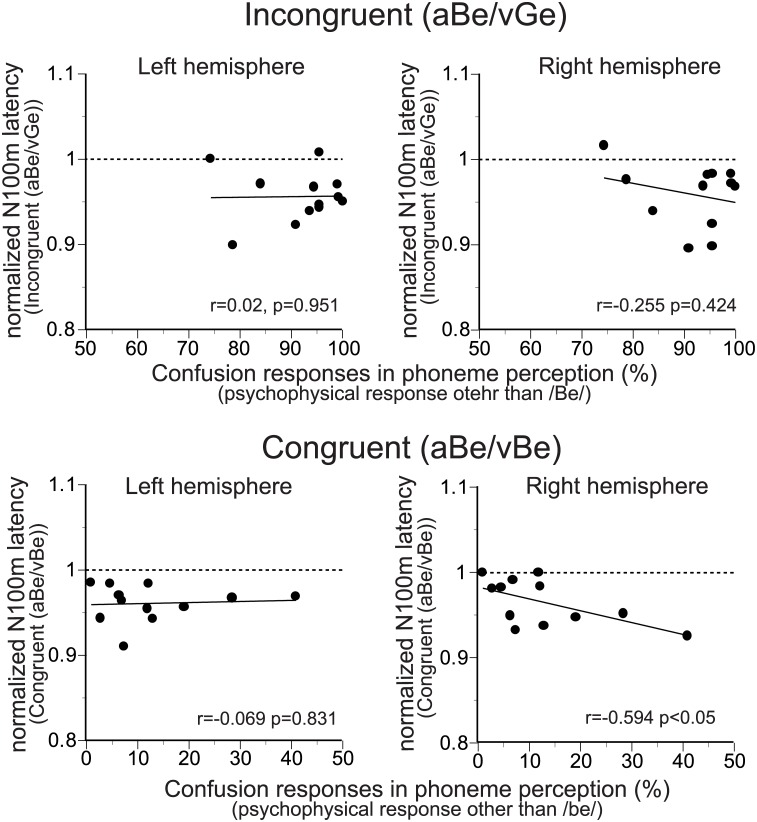
Relationship between the magnitudes of the visual speech effects and psychophysical responses. Normalized N100m latencies (ratio between N100m latencies under the aBe/vBe or aBe/vGe condition and control [aBe/vN] condition) are plotted as a function of confusion responses in phoneme perception (psychophysical responses other than /be/). Theoretically, correlation between psychophysical response and visual speech effects on the N100m latencies should result in positive and negative correlations for incongruent (upper panels) and congruent (lower panels) A/V conditions, respectively. However, no such correlations were observed. Thin line in each figure indicates the linear regression line.

The psychophysical responses during the MEG measurements were also relatively varied among subjects as shown in Fig 2. To investigate the possible relationships between the magnitudes of the psychophysical responses and those of the visual speech effects seen in the N100m latencies, the relationships between normalized N100m latencies and rate of confusion responses in phoneme perception (psychophysical responses other than /be/) were analyzed (Fig 7). Theoretically, correlation between psychophysical response and visual speech effects on the N100m latencies should result in shorter normalized N100m latencies with higher rate of confusion responses in phoneme perception under the incongruent A/V condition. In contrast, normalized N100m latencies should be shorter with lower rate of confusion responses in phoneme perception under the congruent A/V conditions. However, no such correlation was observed.

## Discussion

The present study examined the lip-reading effects of monosyllables on AEFs generated from the auditory cortices. Overall, lip reading had significant effects on the latency reduction of N100m with no apparent hemispheric laterality. However, the degree of N100m shortening was not significantly different between congruent and incongruent A/V conditions, although behavioral data showed psychophysical responses were significantly different between these two A/V conditions. The magnitude of visual speech effects on N100m was varied among subjects, but the lip-reading effects on AEFs tended to be well correlated between the two different A/V conditions (congruent vs. incongruent visual stimuli) in the bilateral hemispheres on an individual basis. On the other hand, no significant correlation was observed between the magnitudes of visual speech effects and psychophysical responses.

The magnitude of the visual effects, as assessed psychophysically, is known to vary among subjects. For example, there are large individual differences in the frequency of the McGurk effect, from 0% to 100% across individuals [18, 27–29], although nearly 100% of adult participants were initially reported to perceive the McGurk illusion [3].

In this context, the present study examined the relationship between the confusion responses in phoneme perception (i.e., other than /Be/) under McGurk stimuli (i.e., aBe/vGe) and the visual effects on N100m latency, but found no apparent positive relationship. Imaging studies have indicated that extensive areas from the primary-secondary auditory cortices to the STS are important in human lip-reading effects among the processing sites of auditory-visual coupling [4–14]. Hemodynamic studies of the individual differences of McGurk effects indicated that the amplitude of the response in the left STS was significantly correlated with the likelihood of perceiving the McGurk effect: a weak STS response meant that a subject was less likely, whereas a strong response meant that a subject was more likely to perceive the McGurk effect [30, 31]. Considering that the N100m is one of the most stable evoked waves mainly originating from the primary-secondary auditory cortices, the A/V interaction seen in the auditory cortex may just be an initial stage, and the psychophysical response as a final judgment of A/V perception would occur at a later processing stage.

On the other hand, visual speech effects on N100m latency were significantly correlated between the two different visual conditions (congruent visual stimuli /be/ vs. incongruent visual stimuli /ge/) in the bilateral hemispheres. The visual effect on N100m could be observed with any type of visual speech presented synchronously with speech sound regardless of congruency between the auditory and visual inputs, but the magnitude of the visual effects was considerably affected by the visual predictability (ease of recognition of the speech information based only on visual clues) [14, 20]. In the present study, in order to observe the relationship of individual variance between the psychophysical and neurophysiological (i.e., N100m) responses, incongruent visual stimuli (visual /ge/ for auditory /be/) expected to evoke the McGurk illusion were used as the visual stimuli in addition to the congruent visual stimuli (visual /be/). The effects of visual /ge/ on N100m latency were reported to be smaller than those of labial consonant [15]. In contrast, the present study found the effects on N100m latency by visual /ge/ were equally large as those by visual /be/. The reason for these somewhat different findings in the previous report and present study are unclear. However, cultural differences between western and Japanese subjects may be related to this difference, since the auditory—magnitude of visual coupling such as McGurk effects were different among subjects with different cultural backgrounds [28, 29]. In any case, it is worth noting that the magnitude of visual effects on N100m latency was well correlated between the two different visual speech stimuli which cause apparently different psychophysical responses. This observation may suggest that the inter-individual differences in processing time for the N1 response did not depend on the stimulus content.

Correlation of visual-speech effects on N100m between the right and left hemispheres was not significant, although visual effects on N100m were not significantly different overall between the right and left hemispheres. Left hemispheric dominancy in human lip-reading effects was indicated at the level of STS [4–10]. Therefore, the insignificant correlation between the right and left hemispheres in visual effects on N100m latency may hint at different functions in auditory-visual coupling in the right and left hemispheres. Nevertheless, the visual effects on N100m were significantly correlated between the two different visual stimuli in each hemisphere, which may indicate that the observed auditory-visual interaction on the N100m is a fundamental process which does not depend on the congruency of the visual information.

## Conclusion

The present study investigated the individual variations in the effects of lip reading on the latencies of N100m as well as psychophysical responses, by examining the lip-reading effects under congruent and incongruent visual conditions. The individual visual-speech effects on N100m latency (shortening of the N100m latency) were significantly correlated between congruent and incongruent A/V stimuli, but did not reflect the individual differences in psychophysical confusion. These findings suggest that the inter-individual difference of processing time for N100m does not depend on stimulus content. Moreover, the A/V interaction seen in the auditory cortex may just be an initial stage, and the psychophysical response as a final judgment of A/V perception occurs at a later processing stage.

## Supporting Information

S1 Data File containing the raw data to replicate Figs 2, 4, 5, 6 and 7. (PDF)
