# Supplementary material for: Effects of Visual Speech on Early Auditory Evoked Fields - From the Viewpoint of Individual Variance
Source: PLoS One. 2017 Jan 31;12(1):e0170166. doi: 10.1371/journal.pone.0170166 (PMC5283660; doi:10.1371/journal.pone.0170166)
Supplement: S1 Data — (PDF) [file pone.0170166.s001.pdf]

1) N100m Latency (raw data)

a) Left hemisphere

N100m latency (ms) Left hemisphere

	aBe/vN	aBe/vBe	aBe/vGe
case 1	112	102	106
case 2	106	100	100
case 3	132	126	124
case 4	122	120	118
case 5	130	126	120
case 6	136	132	132
case 7	136	134	136
case 8	122	118	116
case 9	138	132	134
case 10	111	107	106
case 11	122	120	123
case 12	139	131	125

b) Right hemisphere

N100m latency (ms) Right hemisphere

	aBe/vN	aBe/vBe	aBe/vGe
case 1	118	110	106
case 2	106	104	98
case 3	128	128	124
case 4	110	108	108
case 5	134	124	120
case 6	118	112	116
case 7	122	122	124
case 8	124	118	120
case 9	132	125	124
case 10	106	105	103
case 11	118	116	116
case 12	127	119	124

2) Psychophysical Response (raw data)

a) aBe/vN

aBe/vN	Response (number of times)		(total)
	other than /be/	/be/	
case 1	24	86	110
case 2	9	101	110
case 3	38	72	110
case 4	33	77	110
case 5	68	42	110
case 6	76	34	110
case 7	8	102	110
case 8	61	49	110
case 9	71	61	132
case 10	19	112	131
case 11	54	78	132
case 12	41	91	132

b) aBe/vBe

aBe/vBe	Response (number of times)		(total)
	other than /be/	/be/	
case 1	8	102	110
case 2	3	106	109
case 3	13	97	110
case 4	5	105	110
case 5	45	65	110
case 6	7	103	110
case 7	1	109	110
case 8	31	78	109
case 9	25	106	131
case 10	9	123	132
case 11	16	116	132
case 12	17	115	132

c) aBe/vGe

aBe/vGe	Response (number of times)		(total)
	other than /be/	/be/	
case 1	105	5	110
case 2	105	5	110
case 3	103	7	110
case 4	103	6	109
case 5	100	10	110
case 6	109	1	110
case 7	81	28	109
case 8	110	0	110
case 9	110	21	131
case 10	130	1	131
case 11	126	6	132
case 12	103	28	131
